# Secondary antibody deficiency: a complication of anti-CD20 therapy for neuroinflammation

**DOI:** 10.1007/s00415-018-8812-0

**Published:** 2018-03-06

**Authors:** E. C. Tallantyre, D. H. Whittam, S. Jolles, D. Paling, C. Constantinesecu, N. P. Robertson, A. Jacob

**Affiliations:** 1 University Hospital of Wales, Cardiff, UK; 2 Cardiff University School of Medicine, Cardiff, UK; 3 The Walton Centre NHS Trust, Liverpool, L97LJ UK; 4 University of Liverpool, Liverpool, UK; 5 NIHR Sheffield Biomedical Research Centre (Translational Neuroscience), Sheffield, UK; 6 Royal Hallamshire Hospital, Sheffield, UK; 7 University of Nottingham, Nottingham, UK

**Keywords:** Anti-CD20, Rituximab, Secondary antibody deficiency, Infection, Complication

## Abstract

B-cell depleting anti-CD20 monoclonal antibody therapies are being increasingly used as long-term maintenance therapy for neuroinflammatory disease compared to many non-neurological diseases where they are used as remission-inducing agents. While hypogammaglobulinaemia is known to occur in over half of patients treated with medium to long-term B-cell-depleting therapy (in our cohort IgG 38, IgM 56 and IgA 18%), the risk of infections it poses seems to be under-recognised. Here, we report five cases of serious infections associated with hypogammaglobulinaemia occurring in patients receiving rituximab for neuromyelitis optica spectrum disorders. Sixty-four per cent of the whole cohort of patients studied had hypogammaglobulinemia. We discuss the implications of these cases to the wider use of anti-CD20 therapy in neuroinflammatory disease.

## Introduction

B-cell-depleting anti-CD20 monoclonal antibody therapies have demonstrated significant clinical efficacy in individuals with neuroinflammatory disorders and are increasingly gaining traction as a therapeutic approach. In particular, open label data consistently demonstrate favourable efficacy for rituximab (a chimeric anti-CD20 monoclonal antibody) on clinical outcomes in patients with neuromyelitis optica (NMO) [1] as well as a reduction in MRI and clinical measures of disease activity in phase-II studies of relapsing multiple sclerosis (MS) [2–4]. More recently, ocrelizumab (a fully humanised anti-CD20 monoclonal) has also been shown to be effective in relapsing MS and is the first therapeutic agent to have exhibited a reduction in disability progression in a phase-III study of primary progressive MS [5, 6]. It has gained FDA approval and is expected to become a key drug for MS globally in the next few years.

Anti-CD20 monoclonal antibody therapy is typically used as maintenance therapy when it is prescribed for NMO or MS. This is in contrast to its widespread use in many non-neurological diseases including rheumatoid arthritis and anti-neutrophil cytoplasmic antibody (ANCA)-associated vasculitis, in which anti-CD20 therapy is usually employed as a short-term remission-inducing agent before the subsequent introduction of an alternative maintenance therapy [7]. While the use of maintenance anti-CD20 therapy has increased significantly in neurology, long-term safety data on its use in neuroinflammatory disease remain scarce.

A profound and durable depletion of circulating B cells occurs within days of rituximab infusion [8, 9]. The fact that plasma cells do not express CD20 and are, therefore, resistant to the immediate depletory effects of anti-CD20 therapy is expected to preserve humoral immunity [10], but secondary antibody deficiency does occur [9, 11–14]. However, its frequency or consequences in neurological diseases have not been fully explored.

Here, we present a UK cohort of 50 individuals with NMO treated over extended periods with rituximab, five of whom developed serious infection in the context of secondary antibody deficiency. We consider the implications of this case series and outline potential considerations in the long-term use of anti-CD20 monoclonal antibodies in NMO and other neuroinflammatory diseases.

### Patient identification and acquisition of laboratory data

Management of individuals with NMO in England is coordinated by the Walton Centre, Liverpool, and John Radcliffe Hospital, Oxford. Patients in Wales are referred to regional specialist services, including Cardiff. Disease-specific databases and detailed longitudinal clinical data are available in all centres. Interrogation of the Liverpool and Cardiff datasets was used to identify any individual with a diagnosis of NMO or NMO spectrum disorder (NMO-SD) who had been treated with rituximab since 2007 (Liverpool *n* = 50, Cardiff *n* = 6). Results of laboratory investigations including serum immunoglobulins were known for 50 out of 56 patients. Review of case records was undertaken in a subset of patients known to have experienced severe infection (defined as requiring hospital admission and IV antibiotics) in the context of hypogammaglobulinaemia (reduced blood concentration of IgG, IgM and/or IgA).

All patients provided consent for their data to be used as part of ethically approved research studies (reference numbers 15/L0/1433 and 05/WSE03/111). Immunoglobulin levels (IgG, IgA and IgM) were assayed by nephelometry (Siemens BN2 Nephelometer; Siemens) and specific antibody titres against *Haemophilus influenzae* (Hib), *Clostridium tetani* (tetanus) and Pneumococcal capsular polysaccharide were determined by ELISA (The Binding Site, Birmingham, UK). Protective cut-off levels of circulating specific antibodies were tetanus > 0.1 IU/ml, *Haemophilus influenzae* > 1mcg/ml and Pneumococcal antibodies > 50 mg/L [15].

A total of 33 archived serum samples were available, from disease-specific biobanks, from the five cases with severe infection. Samples were analysed for immunoglobulin levels over 14.6 patient-years (mean 2.7 samples per patient per year). A total of 19 samples were analysed for disease-specific Igs over 15.3 patient-years (mean 1.3 samples per patient per year).

## Results

### Serum immunoglobulins

Immunoglobulin levels were available for 50 patients at some time point. Baseline (pre-rituximab) values were available for 23 cases. Four of the 23 cases in whom sera were available (8% of the cohort) were recorded to have low baseline IgG (< 6.0 g/L) and a further 15 out of 50 (30%) patients were recorded as developing IgG deficiency at some stage during rituximab therapy. Overall, 28 patients (56%) were detected to have IgM deficiency (< 0.4 g/L) at some stage during rituximab therapy (one of whom had low levels at baseline) and 9 (18%) were found to have IgA deficiency (< 0.8 g/L) during rituximab therapy (one of whom had low levels at baseline). The mean nadir of IgG was 4.5 g/L recorded after a mean of 3.4 years of rituximab therapy (median 3.5, range 0.2–8.9). Overall 18 out of 50 (36%) patients receiving rituximab were not recorded to have immunoglobulin deficiency (mean follow-up duration 3.2 years).

### Cases of severe infection

**Fig. 1:**
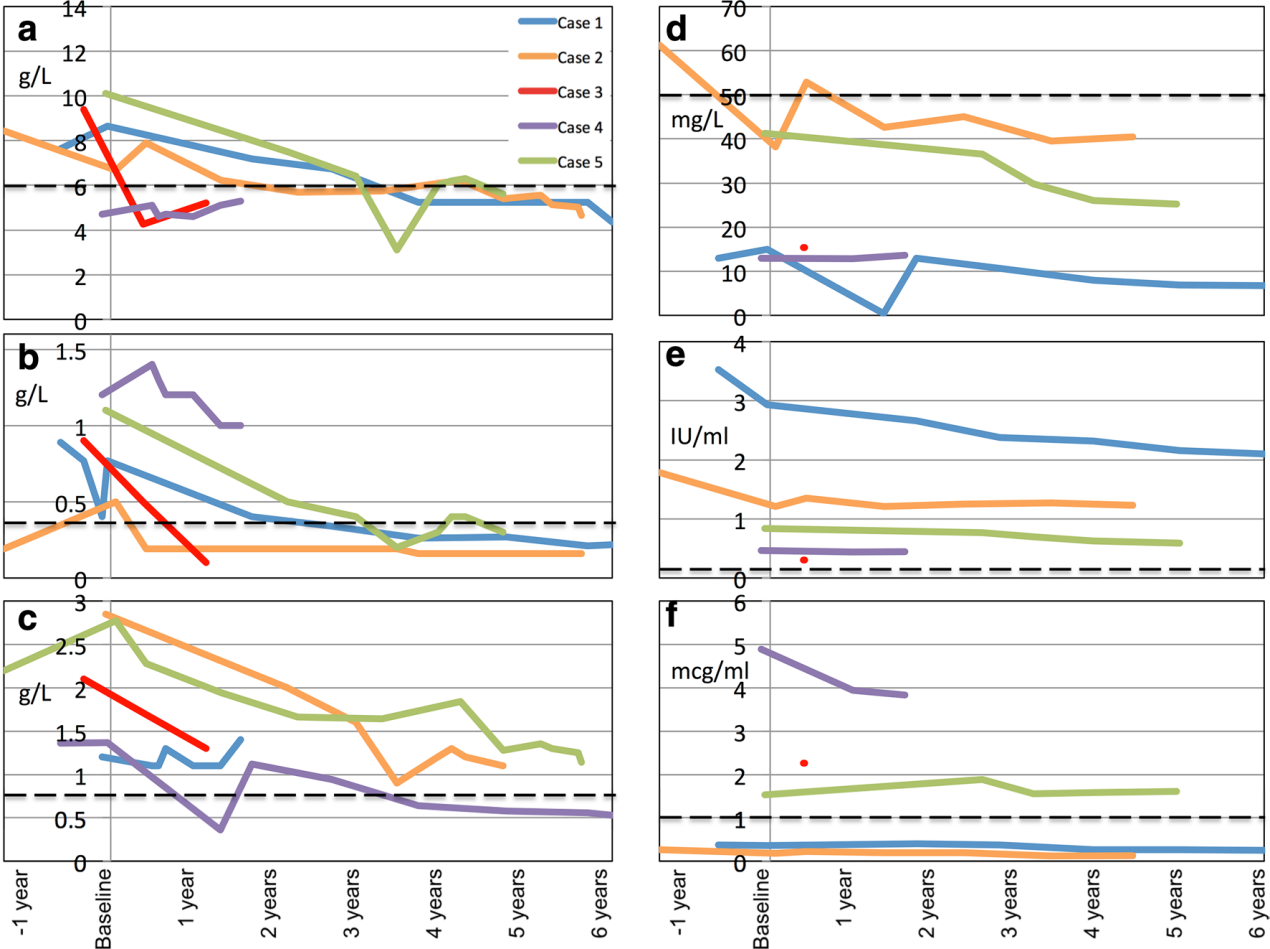
Serial immunoglobulins measured in the five individuals with hypogammaglobulinemia and severe infection. In each case, serial measurements are plotted on the y-axis, against time from the commencement of rituximab (baseline) on the x-axis. Dashed black lines indicate the lower levels of the normal ranges. Data were censored at time of immunoglobulin replacement therapy where relevant. **a** IgG, **b** IgM, **c** IgA, **d** Anti-pneumococcal antigen antibodies. **e** Anti-tetanus antibodies, **f** Anti-HiB antibodies

Five out of 50 cases were identified had experienced severe infection in the context of low serum Ig. All were female and all had aquaporin-4 antibody-positive NMO. Clinical and demographic features are shown in Table 1. Serial immunoglobulin data are shown in Fig. 1.

**Table 1:** Clinical and demographic features of five individuals who experienced severe infection and hypogammaglobulinaemia during rituximab therapy

	Age at disease onset (years)	Cumulative duration and type of DMT prior to RTX (in order given)	Duration of disease at RTX onset (year)	EDSS at RTX onset	Baseline IgG (g/L)*	RTX treatment schedule	ARR pre- RTX	ARR post-RTX	Time from RTX to detection of low IgG (years)	RTX duration and cumulative dose at time of severe infection	Igs at time of severe infection (low titres in bold)*	Nature of severe infection	Ongoing management
Case 1	9	3.3 years; β interferon, PLEx, Aza	22	7.5	8.7 (pre-RTX)	CD19-led then 6-monthly after 3 years	0.6	0.1	3.8	6.2 y, 11 g	IgG **4.4** IgM **0.2** IgA **0.5** HiB **0.25** Pneum **6.7** Tet 2.1	Bronchiectasis, suppurative otitis media, mycoplasma pneumonia	Regular IGRT (3-weekly) Prophylactic abx Back-up abx
Case 2	36	0.8 years; Aza, MMF	14	6.5	6.7 (pre-RTX)	6-monthly	0.5	0.2	2.3	4.5y, 12 g	IgG **5.4** IgM **< 0.2** IgA 1.3 HiB **0.13** Pneum **40** Tet 1.2	Bronchiectasis and sepsis, presumed urinary	Regular IGRT Prophylactic abx Back-up abx
Case 3	53	0.3 years; MMF, Aza	0.4	6.0	9.4 (pre-RTX)	2 g every 6 months. Concurrent prednisolone for first 12 months of RTX.	5.1	0.9	0.4	0.9y, 5 g	IgG **5.2** IgM **< 0.2** IgA 1.3 HiB 2.3 Pneum **15** Tet 0.3	Legionella pneumonia	Ig monitoring Back-up abx
Case 4	39	11.2 years; Mitox, Aza, MMF, Aza	18	7.0	4.7 (pre-RTX)	Two 2 g infusions, 8 months apart, then CD19-led 1 g retreatments. Concurrent prednisolone for duration of RTX treatment.	0.73	0.6	Low at baseline	0.7y, 5 g	IgG **4.7** IgM **1.2** IgA 1.3 HiB 3.9 Pneum **13** Tet 0.4	Pneumonia	N/a (deceased)
Case 5	15	2.0 years; Aza, MMF	3	4.0	10.1 (pre-RTX)	2 g every 6 months for 3y then CD19-led. Concurrent prednisolone for first 3 years of RTX.	2.2	0	3.5	3.5y, 14 g	IgG **3.1** IgM **0.2** IgA 0.9 HiB 1.6 Pneum **30** Tet 0.7	Pneumonia	Switched to CD19-led infusions and IgG normalised. Discontinued prophylactic abx thereafter.

*Abx* antibiotics, *ARR* annualised relapse rate, *Aza* azathioprine, *EDSS* Expanded disability status scale, *HiB* anti-*haemophilus influenza antibodies*, *Igs* immunoglobulins, *mitox* mitoxantrone, *MMF* mycophenolate mofetil, *PLEx* plasma exchange, *pneumo* anti-pneumococcal antibodies, *RTX* rituximab, *Tet* anti-tetanus antibodies, *IGRT*- Immunoglobulin Replacement Therapy

*Normal ranges: IgG 6–16 g/L, IgM 0.4–2.5 g/L, IgA 0.8–3 g/L, HiB > 1mcg/ml, Pneum > 50 mg/L, Tet > 0.1 IU/ml

#### Case 1

Presented in 1987, aged 9 years with intractable vomiting and left optic neuritis. Her MRI brain showed multiple areas of T2 hyperintensity consistent with CNS demyelinating disease and she had unpaired CSF oligoclonal bands. She initially received a diagnosis of MS and had 1 year of beta-interferon therapy. She went on to experience three further episodes of optic neuritis and at least five episodes of myelitis, each followed by poor recovery despite intravenous steroids. By 2006, when the anti-AQP4 antibody had become widely available and she tested positive; at that time she was registered blind with an EDSS of 6. She had 6-monthly plasma exchange for 18 months following 6 months of azathioprine. Side-effects and evidence of ongoing CNS inflammatory activity prompted consideration of rituximab. Baseline (pre-rituximab) EDSS was 7.5. Baseline immunoglobulins were normal; however, IgG was in the lower half of the normal range and anti-pneumococcal and anti-HiB antibodies were both low (tested in retrospect using biobanked serum) (Fig. 1). In retrospect, using archived serum samples, low IgG was first detectable after 3.6 years of rituximab. After 6.2 years of rituximab, she presented with chronic cough and was found to have bilateral bronchiectasis on CT chest imaging in the absence of a smoking history. She was concurrently being investigated for hearing loss and was found to have bilateral suppurative otitis media with perforation of both tympanic membranes. At the time, serum IgG was 4.4 g/L (normal range 6–16), IgA 0.5 g/L (normal range 0.8–3) and IgM 0.2 (normal range 0.4–2.5). Shortly after the diagnosis of bronchiectasis, she developed superimposed *Mycoplasma pneumoniae* infection necessitating several weeks in an intensive care unit (ICU). She recovered from her pneumonia although she remains severely hearing impaired. She elected to continue with 6-monthly rituximab infusions but now receives additional 3-weekly immunoglobulin replacement therapy (IGRT), which has significantly decreased her infection burden. Her EDSS has remained stable (7.5) during 9 years of rituximab therapy.

#### Case 2

Presented in 2006, aged 45y with bilateral, sequential optic neuritis followed by poor visual recovery. She had a previous history of arm and leg weakness compatible with myelitis some years earlier. Investigations ultimately fulfilled diagnostic criteria for AQP4 + NMO in 2009. She was commenced on azathioprine but experienced breakthrough disease activity and deranged liver function during 3 months of mycophenolate therapy. By the time she commenced rituximab therapy in 2011, EDSS had reached 7. She had low baseline IgM and low anti-HiB antibodies (tested in retrospect using biobanked serum). In retrospect, using archived serum samples, low IgG was first detectable after 2.3 years of rituximab. After 4.5 years of rituximab therapy, she developed chronic cough, in the absence of a smoking history, which persisted despite several courses of antibiotics. Sputum culture revealed a heavy growth of *Haemophilus influenzae*. Chest X-rays were normal but she was found to have bilateral bronchiectasis on high-resolution CT chest imaging. She had monitoring of immunoglobulin levels, received antibiotic prophylaxis and a course of back-up antibiotics for breakthrough respiratory infective symptoms. She was vaccinated against pneumococcus and HiB but subsequent disease-specific antibody measurements showed that she did not mount a protective response. She elected to continue with 6-monthly rituximab therapy, having experienced 4 years of relapse freedom. Despite preventative measures, she developed an episode of septicaemia due to urinary tract infection in 2017. IgG at the time was 5.1 g/L, IgM < 0.17 g/L and IgA normal. She was in hospital for 11 days, requiring fluid resuscitation, IV antibiotics and IGRT. Since recovering from her most recent infection, she has elected to continue with rituximab therapy with additional regular IGRT. Her current EDSS is 7.5.

#### Case 3

Presented in 2015, aged 53y with longitudinally extensive transverse myelitis and was found to meet diagnostic criteria for AQP4 + NMO. She was treated briefly with mycophenolate in 2015 but switched to azathioprine due to gastric ulceration. She commenced rituximab in 2016, when her EDSS was 6. Her baseline IgG was in the lower half of the normal range. Baseline disease-specific antibodies are unavailable. She was treated with 6-monthly rituximab infusions with concurrent prednisolone for the first 12 months. Low serum IgG (tested in retrospect using biobanked serum) was first detectable after 4.5 months of rituximab. She developed *Legionella* pneumonia after 11 months of rituximab treatment, necessitating invasive ventilation on ICU. She recovered from her pneumonia and elected to continue with 6-monthly rituximab therapy. Her hypogammaglobulinaemia has persisted and despite vaccination against pneumococcus and HiB, she has continued to experience recurrent chest infections requiring frequent courses of antibiotics and is planned for IGRT.

#### Case 4

Presented with a brainstem syndrome in 1997, aged 39y and experienced subsequent recurrent optic neuritis and myelitis. She was diagnosed clinically with NMO in 2004 and confirmed to have AQP4 antibodies in 2008. She had chronic obstructive airways disease and continued to smoke cigarettes. She received a single pulse of mitoxantrone in 2004 and then commenced azathioprine and prednisolone. She was switched briefly to mycophenolate in 2014 but commenced rituximab in 2015 because of side-effects and breakthrough disease activity on previous agents. At the time her EDSS was 7.5. Baseline IgG levels and anti-pneumococcal antibodies were both low. She continued prednisolone (daily dose 10–15 mg) alongside her rituximab therapy. She developed pneumonia in 2015; at the time her serum IgG was 4.7 g/L (normal IgM and IgA). In early 2016, she commenced antibiotic prophylaxis and rituximab infusions were scheduled according to B-cell repopulation. Despite these measures, she continued to experience recurrent pneumonia over a 12-month period and ultimately died from her infection in late 2016.

#### Case 5

Developed optic neuritis in 2009, aged 15y. She experienced further episodes of optic neuritis and myelitis, and was found to fulfil diagnostic criteria for NMO in 2010. She was commenced on azathioprine and switched to mycophenolate in 2011 and then to rituximab in 2012 because of breakthrough disease activity. At the time of commencing rituximab her EDSS was 4.0 and serum Igs (including disease-specific antibodies) were normal. She continued to receive daily prednisolone for the first 3 years of rituximab therapy. In 2015 she developed sinusitis and chest infections, which were initially managed in the community. At that time her serum IgG was 3.1 g/L and IgM was also low (0.2 g/L; IgA normal). Later that year she developed pneumonia and required hospitalisation for IV antibiotics. She recovered from her severe infection and elected to continue with rituximab therapy. Her rituximab schedule was switched from 6-monthly infusions to retreatment according to B-cell repopulation. Serum IgG level normalised and she has not experienced any further infections.

## Discussion

B-cell-depleting anti-CD20 monoclonal antibody therapies demonstrate favourable clinical efficacy for individuals with CNS inflammatory disease but long-term safety data are scarce. Hypogammaglobinaemia was recorded in 64% of patients (32 out of 50) for whom data were available in this UK cohort. Severe infection associated with secondary antibody deficiency occurred in 5 out of 50 (10% of the entire cohort). This value may underestimate the true prevalence, as infection and immunoglobulin data were not systematically surveyed in all patients.

This case series illustrates several additional insights. First, rituximab had sustained clinical efficacy in patients that had not been achieved with prior disease-modifying therapies (mean pre-treatment annualised relapse rate of 1.8 versus 0.4 post-rituximab). This was a key factor influencing the decision to continue using rituximab in all cases, despite the episodes of infection. Second, laboratory and clinical surveillance is required to detect the earliest signs of these treatment-associated infections. Third, a combination of serum immunoglobulin measurement plus disease-specific antibody titres appears optimal to determine risk, maximise the opportunity for preventative measures and avoid the development of bronchiectasis.

A profound and durable depletion of circulating B cells occurs within days of rituximab infusion [8, 9]. As plasma cells do not express CD20, they are unaffected and have hitherto provided some assurance that humoral immunity would be sufficiently preserved [10]. However, it remains unclear whether long-term humoral immunity results entirely from a self-sustaining long-lived plasma cell (LLPC) population that survive for decades, or whether regular replenishment of plasma cells by memory B cells is required every few months [16]. Despite the apparent survival of LLPCs after B-cell depletion [17–19], several clinical studies have observed a dose-dependent reduction in serum immunoglobulins following anti-CD20 therapy [11, 12, 14, 20]. This implies that long-term humoral immunity may be more reliant on replenishment of plasma cells from the B-cell progenitor pool than previously thought.

The finding that B-cell depletion lasts an average of 6 months [21], led to regular 6-monthly dosing of rituximab during many early regimens. However, the considerable inter-individual variation in repopulation time has led several authors to recommend retreatment guided by B-cell repopulation, mainly to avoid risk of neurological relapse in those who repopulate early. The use of CD19 + (a pan B-cell marker) monitoring in NMO was initially suggested but the incidence of NMO relapses occurring even at low levels of CD19 + B-cell repopulation suggests this approach may compromise disease control [9, 11]. The observation that the return of CD27 + (memory B cells) coincides with the return of disease activity in rheumatoid arthritis prompted use of CD27 + B-cell monitoring to guide retreatment of NMO [11, 22]. Retreatment guided by CD27 + repopulation has been reported to lower the cumulative dose of rituximab while maintaining remission in the majority of patients (91 out of 100 cases) but is not universally available in clinical laboratories [11, 23].

These data may have implications for use of similar B-cell depleting drugs, notably the newly approved ocrelizumab for MS. Despite considerable inter-individual variation observed in time-to-repopulation of B cells following ocrelizumab [24], dosing is recommended to occur at regular 6-monthly intervals [25]. No data were reported on immunoglobulin levels in phase-III ocrelizumab studies in MS but there was no excess of serious infection in ocrelizumab-treated patients during the 96 weeks of follow-up [6, 25]. It is possible that the risk of secondary antibody deficiency may be higher in an NMO population where other immunosuppressive agents often precede anti-CD20 therapy. The subnormal IgG and pneumococcal antibodies observed pre-rituximab in case 4 (who had already received mitoxantrone, azathioprine and mycophenolate over 10 years) highlight the possible contribution of prior immunotherapy to secondary antibody deficiency. A review of risk factors predisposing to the development of hypogammaglobulinaemia and infections post-rituximab identified low immunoglobulins prior to treatment, maintenance therapy with rituximab, prior immunosuppression, concomitant purine analogues such as mycophenolate and chronic lung or heart disease as well as older age [26]. In four out of five of our cases, the combined duration of previous immunosuppressive therapy and rituximab treatment exceeded 5 years, suggesting that secondary antibody deficiency may be a dose- and duration-dependent phenomenon. Therefore, it is possible that other CD-20-depleting drugs including ocrelizumab may with long-term treatment have similar effects. However, case 3 had received only 4 months of immunosuppression and 11 months of rituximab therapy before developing severe infection associated with secondary antibody deficiency. Possible explanations for the early development of severe infection in this case include the co-administration of maintenance corticosteroid.

The occurrence of recurrent or complicated infections of the upper and/or lower respiratory tract in our case series is typical of antibody deficiency and illustrates the need for surveillance that extends beyond blood monitoring [27]. Monitoring of patients on anti-CD20 therapy should include regular questioning on infective burden with a particular focus on sino-pulmonary symptoms.

In retrospect, using archived sera, we were able to demonstrate downward trends in serum immunoglobulin levels that occurred after the commencement of rituximab. Due to low awareness of this complication of B-cell-depleting therapies, serum immunoglobulin surveillance was not routinely undertaken in this series until recently, leading to delays in detection and treatment in some cases. However, the hypogammaglobulinaemia that coincided with severe infection was often modest and the presence of subnormal baseline protective levels of disease-specific antibodies to HiB and/or pneumococcus suggests that they may add additional functional information to help stratify for the risk of infection during anti-CD20 therapy. Test vaccination as used in the assessment of humoral immunodeficiency may also play a role.

Screening of patients before rituximab therapy for serum immunoglobulins and disease-specific antibodies opens up the possibility for therapeutic vaccination, which is more challenging once B cells are depleted. Detection of on-therapy secondary antibody deficiency ought to prompt close infection surveillance and consideration of back-up or prophylactic antibiotics and/or IGRT to prevent infection and end organ damage. If further pooled long-term data confirm secondary antibody deficiency as a prevalent complication, management algorithms allied to those used for primary antibody deficiency may be required [27].

In summary, this case series highlights a serious complication of anti-CD20 depletion therapy when used as remission maintenance treatment for neuroinflammatory disease. While NMOSD is a relatively rare disease, this observation is potentially generalisable to other diseases treated with B-cell depletion. It is particularly important in the ocrelizumab era of MS treatment. Progressive MS patients who may benefit from ocrelizumab may have additional comorbidities that predispose them to infections. Heightened awareness of this potentially preventable and treatable complication is crucial to avoid added morbidity and mortality and to allow patients to continue benefiting from a highly effective mode of therapy.
