# Correction to: Secondary antibody deficiency: a complication of anti-CD20 therapy for neuroinflammation

**DOI:** 10.1007/s00415-018-8833-8

**Published:** 2018-04-07

**Authors:** E. C. Tallantyre, D. H. Whittam, S. Jolles, D. Paling, C. Constantinescu, N. P. Robertson, A. Jacob

**Affiliations:** 1 University Hospital of Wales, Cardiff, UK; 2 Cardiff University School of Medicine, Cardiff, UK; 3 The Walton Centre NHS Trust, Liverpool, L97LJ UK; 4 University of Liverpool, Liverpool, UK; 5 NIHR Sheffield Biomedical Research Centre (Translational Neuroscience), Sheffield, UK; 6 Royal Hallamshire Hospital, Sheffield, UK; 7 University of Nottingham, Nottingham, UK

## Correction to: Journal of Neurology 10.1007/s00415-018-8812-0

In the original article, the co-author’s family name has been published incorrectly. The correct family name should be Constantinescu.

## References

[1] Genzyme (2016). Lemtrada [prescribing information].

[2] Cohen JA, Coles AJ, Arnold DL (2012). CARE MS I Alemtuzumab versus interferon beta 1a as first-line treatment for patients with relapsing-remitting multiple sclerosis: a randomised controlled phase 3 trial. Lancet.

[3] Coles AJ, Twyman CL, Arnold DL (2012). CARE MS II Alemtuzumab for patients with relapsing multiple sclerosis after disease-modifying therapy: a randomised controlled phase 3 trial. Lancet.

[4] Havrdova E, Arnold DL, Cohen JA (2017). Alemtuzumab CARE-MS I 5-year follow-up: durable efficacy in the absence of continuous MS therapy. Neurology.

[5] Coles AJ, Cohen JA, Fox E (2017). Alemtuzumab CARE-MS II 5-year follow-up: efficacy and safety findings. Neurology.

[6] Al-Sawaf O, Fischer K, Herling CD (2017). Alemtuzumab consolidation in chronic lymphocytic leukaemia: a phase I/II multicentre trial. Eur J Haematol.

[7] Osterborg A, Foa R, Bezares RF (2009). Management guidelines for the use of alemtuzumab in chronic lymphocytic leukemia. Leukemia.

[8] Sylvan ES, Lundin J, Ipek M (2014). Alemtuzumab (anti-CD52 monoclonal antibody) as single-agent therapy in patients with relapsed/refractory chronic lymphocytic leukaemia (CLL)-a single region experience on consecutive patients. Ann Hematol.

[9] Morgan RD, O’Callaghan JM, Knight SR (2012). Alemtuzumab induction therapy in kidney transplantation: a systematic review and meta-analysis. Transplantation.

[10] Van der Zwan M, Baan CC, Van Gelder T (2018). Review of the clinical pharmacokinetics and pharmacodynamics of alemtuzumab and its use in kidney transplantation. Clin Pharmacokinet.

